# CHK1 monitors spindle assembly checkpoint and DNA damage repair during the first cleavage of mouse early embryos

**DOI:** 10.1111/cpr.12895

**Published:** 2020-09-10

**Authors:** Jia‐Qian Ju, Xiao‐Han Li, Meng‐Hao Pan, Yao Xu, Ming‐Hong Sun, Yi Xu, Shao‐Chen Sun

**Affiliations:** ^1^ College of Animal Science and Technology Nanjing Agricultural University Nanjing China

## Abstract

**Objectives:**

DNA damage and errors of accurate chromosome segregation lead to aneuploidy and foetal defects. DNA repair and the spindle assembly checkpoint (SAC) are the mechanisms developed to protect from these defects. Checkpoint kinase 1 (CHK1) is reported to be an important DNA damage response protein in multiple models, but its functions remain unclear in early mouse embryos.

**Materials and Methods:**

Immunofluorescence staining, immunoblotting and real‐time reverse transcription polymerase chain reaction were used to perform the analyses. Reactive oxygen species levels and Annexin‐V were also detected.

**Results:**

Loss of CHK1 activity accelerated progress of the cell cycle at the first cleavage; however, it disturbed the development of early embryos to the morula/blastocyst stages. Further analysis indicated that CHK1 participated in spindle assembly and chromosome alignment, possibly due to its regulation of kinetochore‐microtubule attachment and recruitment of BubR1 and p‐Aurora B to the kinetochores, indicating its role in SAC activity. Loss of CHK1 activity led to embryonic DNA damage and oxidative stress, which further induced early apoptosis and autophagy, indicating that CHK1 is responsible for interphase DNA damage repair.

**Conclusions:**

Our results indicate that CHK1 is a key regulator of the SAC and DNA damage repair during early embryonic development in mice.

## Graphical abstract

Diagram of the roles of CHK1 on the mouse early embryo development. CHK1 plays roles on both DNA repair and spindle assembly checkpoint during the early cleavage of mouse embryos.

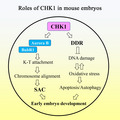

## 1 INTRODUCTION

Mouse embryonic development occurs through a series of cleavage divisions during development from the zygote to the blastocyst stage.^1^ The first cleavage is characterized by a long duration compared with subsequent cleavages. After prolonging the first mitotic cell cycle, the embryo enters a series of rapid divisions, during which the number of cells increases without significant cell growth.^2^ Pre‐implantation embryos can acquire aneuploidy at every developmental stage, but the first two divisions are more susceptible to chromosomal aberrations,^3^ and studies have shown that embryos have higher levels of chromosomal abnormalities in the cleavage stage than in the blastocyst stage.^4^ If there is an error in the replication and separation of genetic information during this process, it will cause serious consequences, such as failure to implant, spontaneous abortion, genetic disease or embryo death.^5^ Cell cycle checkpoints play a crucial role regulating the cell cycle during early embryonic development in mice,^6^ as they monitor the sequence, integrity and fidelity of the main cell cycle events. One of the most important checkpoints is the DNA damage checkpoint. When the DNA damage response (DDR) affects cell proliferation, it reversibly prevents the cell cycle process to allow DNA repair, and the checkpoint is turned off to allow resumption of cell cycle upon completion of DNA repair.^7^ Multiple signal transduction events are coordinated during this process, and the two key of which are the ataxia telangiectasia–mutated/checkpoint kinase 2 and ataxia telangiectasia–mutated and Rad3‐related kinase (ATR)/checkpoint kinase 1 (CHK1) pathways. Activation of these pathways is essential for proper coordination of the checkpoints and DNA repair processes.^8^ DNA damage checkpoints play a vital role ensuring the normal development of embryos, the integrity and stability of DNA, and the generation of healthy offspring. The damage that the embryo inherits from the gametes is repaired before the first mitotic S phase, thereby eliminating the risk of mutagenesis and the disorder during cell differentiation and development of the zygote.^9^ Another checkpoint is the spindle assembly checkpoint (SAC), which causes metaphase arrest when kinetochore‐microtubules are unattached during mitosis.^10^ The SAC consists of ‘sensor’ proteins, such as Mad1, Bub1 and Mps1; a ‘signal transducer’, consisting of the mitotic checkpoint complex, composed of Mad2, Bub3, BubR1 and Cdc20; and an ‘effector’ known as the anaphase promoting complex/cyclosome.^11^ Several lines of evidences indicate that the SAC plays a key role in the mitotic process of early embryonic cells. Deletion of SAC components (such as Bub3, BubR1 and Mad2) accelerates the metaphase‐anaphase transition during the first cleavage in mouse embryos, leading to micronuclei formation, chromosome misalignment and aneuploidy, which decreases implantation and delays development.^12^, ^13^, ^14^, ^15^

The protein kinase CHK1 is a well‐known signal transducer of DNA damage checkpoints, and there is evidence that CHK1 also plays a key role in the spindle checkpoint.^16^ ATR and its binding partner ATRIP are activated when single‐stranded DNA is created at sites of DNA damage or stressed replication forks. CHK1 is a key downstream regulator of the ATR response and is phosphorylated by ATR on Ser‐317 and Ser‐345. Activated CHK1 triggers the intra‐S‐ and G2/M‐phase checkpoints.^17^ In addition, CHK1 negatively regulates Treslin, a TopBP1‐binding protein, to inhibit the initiation of DNA replication.^18^ CHK1‐deficient mice exhibit abnormal cell cycle checkpoint function and early embryo death.^19^ CHK1 is involved in the regulation of the SAC. CHK1‐depleted cells display metaphase block, chromosome misalignment during metaphase, chromosome lag during anaphase and kinetochore defects, which are caused by negative regulation of Plk1 by CHK1.^20^ CHK1 is also required for the SAC by phosphorylating Aurora B and mediating phosphorylation and kinetochore localization of BubR1.^21^ Moreover, CHK1 phosphorylates Mad2 at some sites, particularly S185 and T187,^22^ and CHK1 is also required for the metaphase‐anaphase transition by regulating the subcellular localization and expression of Cdc20 and Mad2.^23^ Decreased CHK1 activity leads to hyper‐stable kinetochore‐microtubules, unstable binding of MCAK, Kif2b and Mps1 to centromeres or kinetochores and reduced phosphorylation of Hec1 by Aurora B.^24^

Although the roles of CHK1 have been reported in several models, its roles during early mouse embryonic development remain unknown. In this study, we used a specific CHK1 inhibitor (Rabusertib) to study the function of CHK1 during mouse early embryo development. Our results indicate that loss of CHK1 activity not only induces oxidative stress and apoptosis, but also causes spindle assembly and chromosome alignment defects, indicating the critical roles of CHK1 during early cleavage of mouse embryos.

## 2 MATERIALS AND METHODS

### 2.1 Antibodies and chemicals

The CHK1 inhibitor Rabusertib was purchased from MedChemExpress. Sheep polyclonal anti‐BubR1 antibody, rabbit monoclonal anti‐γ‐H2A.X and anti‐MAP1LC3A antibodies were obtained from Abcam. The anti‐α‐tubulin‐FITC antibody and Hoechst 33342 were purchased from Sigma. AlexaFluor 488 goat anti‐rabbit antibody and AlexaFluor 594 goat anti‐rabbit antibody were obtained from Invitrogen. The human anti‐centromere CREST antibody was purchased from Fitzgerald Industries International. Mouse polyclonal anti‐BAX, rabbit polyclonal anti‐γ‐H2A.X antibody and anti‐Rad51 antibody were purchased from Proteintech. Rabbit monoclonal anti‐α‐tubulin antibody and phospho‐Aurora A (Thr288)/Aurora B (Thr232)/Aurora C (Thr198) were purchased from Cell Signaling Technology. FITC‐conjugated and TRITC‐conjugated goat anti‐rabbit IgG, TRITC‐conjugated goat anti‐mouse IgG and TRITC‐conjugated donkey anti‐human IgG were obtained from Zhongshan Golden Bridge Biotechnology, Co., Ltd. All other chemicals and reagents were from Sigma‐Aldrich Corp.

### 2.2 Parthenogenetic activation of oocytes and embryo culture

All experiments were approved by the Animal Welfare and Use Committee of Nanjing Agriculture University and were performed in accordance with Animal Research Institute Committee guidelines. To collect embryos, female ICR mice (age, 6‐8 weeks) were stimulated with 5 IU of pregnant mare serum gonadotropin followed 44‐48 hours later by stimulation with 5 IU of human chorionic gonadotropin. After 14 hours, cumulus oocyte complexes were collected from the ampullae of the oviducts and were treated with 10 mg/mL hyaluronidase at 37°C for 5 minutes. The exposed metaphase II oocytes were washed three times in M2 medium and placed in chemical parthenogenetic activation medium (5 μg/mL CB, 2 mmol/L EGTA and 5 mmol/L SrCl_2_) for 5 hours. Then, the zygotes were cultured in M16 medium under paraffin oil at 37°C in a 5% CO_2_ atmosphere.

### 2.3 Rabusertib treatment

Rabusertib was dissolved in DMSO to a 10 mmol/L reserve solution and was diluted with M16 medium to 2.5 and 5 μmol/L working concentrations, respectively, with the final concentration of the solvent not more than 0.1% of the culture medium. The 2.5 μmol/L concentration was eventually used as the treatment in our experiment.

### 2.4 Immunofluorescence microscopy

The embryos were fixed in 4% paraformaldehyde for 30 minutes and then permeabilized with 0.5% Triton X‐100 for 20 minutes at room temperature. After blocking for 1 hour in 1% BSA‐supplemented phosphate‐buffered saline (PBS), the embryos were stained with different primary antibodies/agents (α‐tubulin 1:200; CREST 1:200; LC3 1:100; γ‐H2A 1:200). The embryos were left at room temperature for 8 hours or 4°C overnight. The embryos were further incubated with secondary antibodies (AlexaFluor 488 goat anti‐rabbit or AlexaFluor 594 goat anti‐rabbit antibody; 1:200) for 1 hour at room temperature after washing three times (2 minutes each) in wash buffer (0.1% Tween 20 and 0.01% Triton X‐100 in PBS). Finally, all embryos were stained with Hoechst 33342 (10 mg/mL in PBS) for 10 minutes at room temperature. The samples were fixed on glass slides and examined with a laser‐scanning confocal fluorescent microscope (Zeiss LSM 800 META, Zena).

### 2.5 Reactive oxygen species (ROS) detection

We used a Reactive Oxygen Species Assay Kit (DCFH‐DA; Beyotime Institute of Biotechnology, Beijing, China) to analyse oxidative stress levels in living 2‐cell stage embryos. The live embryos were placed in M16 medium containing DCFH‐DA (1:800) and incubated at 37°C for 30 minutes. We transferred the embryos to preheated fresh M16 and washed them three times. A confocal fluorescent microscope (Olympus CKX53) was used to detect the ROS fluorescent signal. The fluorescence intensities were analysed by ImageJ software (National Institutes of Health).

### 2.6 Annexin‐V staining

An Annexin‐V staining kit (Vazyme Biotech Co, Ltd) was used to detect early apoptosis. Living embryos were placed in M16 medium (Annexin‐V‐FITC 1:10; Hoechst 33342 1:500) for 30 minutes at 37°C. The embryos were fixed in 4% paraformaldehyde for 30 minutes, permeabilized in 0.5% Triton X‐100 for 20 minutes and blocked in 1% BSA‐supplemented PBS at room temperature for 1 hour. The samples were mounted on glass slides and detected with a laser‐scanning confocal fluorescent microscope (Zeiss LSM 800 META).

### 2.7 Western blot analysis

Ninety 2‐cell embryos were placed in Laemmli sample buffer (sodium dodecyl sulphate [SDS] sample buffer with 2‐mercaptoethanol) and heated at 100°C for 10 minutes. The proteins were subjected to 12% SDS‐polyacrylamide gel electrophoresis. After separation, the proteins were transferred to a polyvinylidene fluoride membrane (Millipore) at 20 V for 70 minutes. The membranes were blocked with Tris‐buffered saline (TBS) containing 0.1% (w/w) Tween 20 (TBST) and 5% non‐fat dry milk at room temperature for 1 hour to avoid non‐specific binding, followed by an overnight incubation at 4°C with mouse polyclonal anti‐BAX (1:1000), rabbit monoclonal anti‐α‐tubulin antibody (1:2000), rabbit polyclonal anti‐γ‐H2A.X antibody (1:500) and rabbit monoclonal anti‐Rad51 antibody (1:500). After washing three times in TBST (10 minutes each), the membranes were incubated at 37°C for 1 hour with HRP‐conjugated secondary antibodies (1:2000) in TBST. Finally, the membranes were exposed to an enhanced chemiluminescence reagent (Millipore) and the protein bands were visualized with the Tanon‐3900 instrument.

### 2.8 Quantitative real‐time polymerase chain reaction (qRT‐PCR)

Thirty 2‐cell stage embryos were collected using the Dynabeads mRNA DIRECT kit (Invitrogen Dynal AS) to extract RNA, reversed transcribed to cDNA using the PrimeScript RT Master Mix (Takara) and stored at −20°C until use. Each 20 μL PCR system consisted of 10 µL of Fast Universal SYBR Green Master (ROX); 0.4 µL each of the forward and reverse primers (GAPDH, R:5′‐AGG TCG GTG TGA ACG GAT TTG‐3′, F:5′‐TGT AGA CCA TGT AGT TGA GGT CA‐3′; SOD, R: 5′‐AAA GCG GTG TGC GTG CTG AA‐3′, F: 5′‐CAG GTC TCC AAC ATG CCT CT‐3′; CAT, R: 5′‐GCA GAT ACC TGT GAA CTG TC‐3′, F: 5′‐GTA GAA TGT CCG CAC CTG AG‐3′; P62, R: 5′‐AGG ATG GGG ACT TGG TTG C‐3′, F: 5′‐TCA CAG ATC ACA TTG GGG TGC‐3′; ATG14, R: 5′‐GAG GGC CTT TAC GTG GCT G ‐3′, F: 5′‐ AAT AGA CGA AAT CAC CGC TCT G ‐3′; Rad51, R: 5′‐CGG TGC ATA AGC AAC AGC C‐3′, F:5′‐ AAG TTT TGG TCC ACA GCC TAT TT‐3′; Rad54, R:5′‐GCC GGT TGA GTA GCT GAG TC‐3′, F:5′‐GAC AGT AAC TCC TAA GAA ACG CA‐3′); 1 µL cDNA; and 7.4 µL ddH_2_O. qRT‐PCR was performed using a rapid real‐time PCR system (ABI Step One Plus; ABI). The relative expression level was determined by the 2^−ΔΔCt^ method. The experiment was performed at least three times.

### 2.9 Statistical analysis

At least three replicates were performed for each experiment. The results were presented as mean ± standard error. Statistical comparisons were made using the independent‐sample *t* test and GraphPad Prism 5 software (GraphPad Software Inc). A *P*‐value < .05 was considered significant.

## 3 RESULTS

### 3.1 Loss of CHK1 activity affects early embryonic development in mice

**Figure 1:**
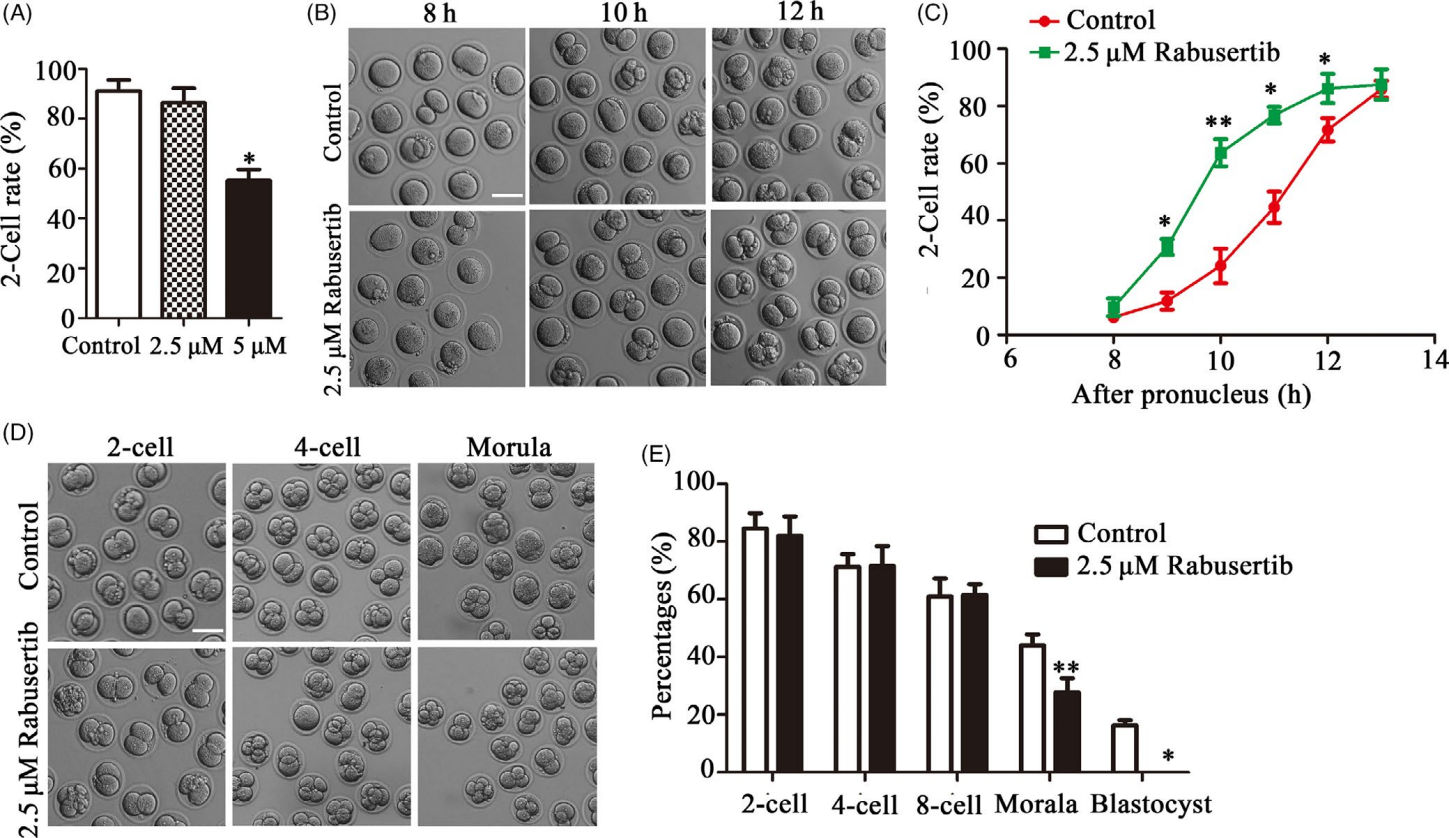
Inhibiting checkpoint kinase 1 (CHK1) activity affects development of early mouse embryos. A, The rates of 2‐cell embryos in the control and 2.5 and 5 μmol/L treatment group embryos. *Significant difference (*P* < .05). B, The cell cycle progression of the first cleavage after inhibiting CHK1 in mouse embryos. Bar = 100 μm. C, The rates of 2‐cell embryos at different time points during the first cleavage in mouse embryos. *Significant difference (*P* < .05) **Significant difference (*P* < .01). D, The developmental rate of early embryos in the treatment group at each stage decreased significantly compared with that in the control group. Bar = 100 μm. E, The developmental rate of early embryos in the control and treatment groups at each stage. *Significant difference (*P* < .05) **Significant difference (*P* < .01)

We used the CHK1 specific inhibitor Rabusertib to explore the potential role of CHK1 during mouse early embryonic development. Rabusertib was used to treat embryos at 2.5 and 5 μmol/L concentrations. As shown in Figure 1A, 2.5 μmol/L Rabusertib did not affect the first cleavage; however, inhibition of CHK1 at the 5 μmol/L concentration significantly reduced the cleavage rate of the embryos (55.22 ± 4.44%, n = 107, 5 μmol/L vs 91.17 ± 4.34%, n = 128, control, *P* < .05; Figure 1A). Although 2.5 μmol/L Rabusertib did not affect cleavage, it accelerated 2‐cell embryo formation: most 1‐cell embryos developed into 2 cells in the control group at 12‐14 hours after the pronucleus formation; however, most of the embryos completed this transition within 9‐11 hours in the 2.5 μmol/L Rabusertib treatment group, suggesting that inhibiting CHK1 accelerated the embryonic cell cycle (Figure 1B). The statistical data confirmed this finding (9 hours: 30.71 ± 2.71%, n = 124 vs 11.81 ± 3.00%, n = 136, *P* < .05; 10 hours: 63.66 ± 4.71%, n = 124 vs 24.09 ± 6.08%, n = 136, *P* < .01; 11 hours: 76.84 ± 2.91%, n = 124 vs 44.64 ± 5.45%, n = 136, *P* < .05; 12 hours: 86.15 ± 5.14%, n = 124 vs 72.94 ± 4.09%, n = 136, *P* < .05) (Figure 1C). In contrast, we continued to culture the embryos and detected developmental defects in embryos from the 2.5 μmol/L Rabusertib treatment group. The results showed that most of the treated embryos did not develop to the morula stage (44.01 ± 3.78% n = 119 vs 27.77 ± 4.86% n = 121, *P* < .01; Figure 1E), and almost none of the embryos developed to the blastocyst stage in the treatment groups, compared with 16% in the control group (Figure 1D,E). These findings indicated that inhibiting CHK1 accelerated the cell cycle and first cleavage but affected subsequent development of early mouse embryos.

### 3.2 Inhibiting CHK1 affects spindle morphology and chromosome alignment at the first cleavage of mouse embryos

**Figure 2:**
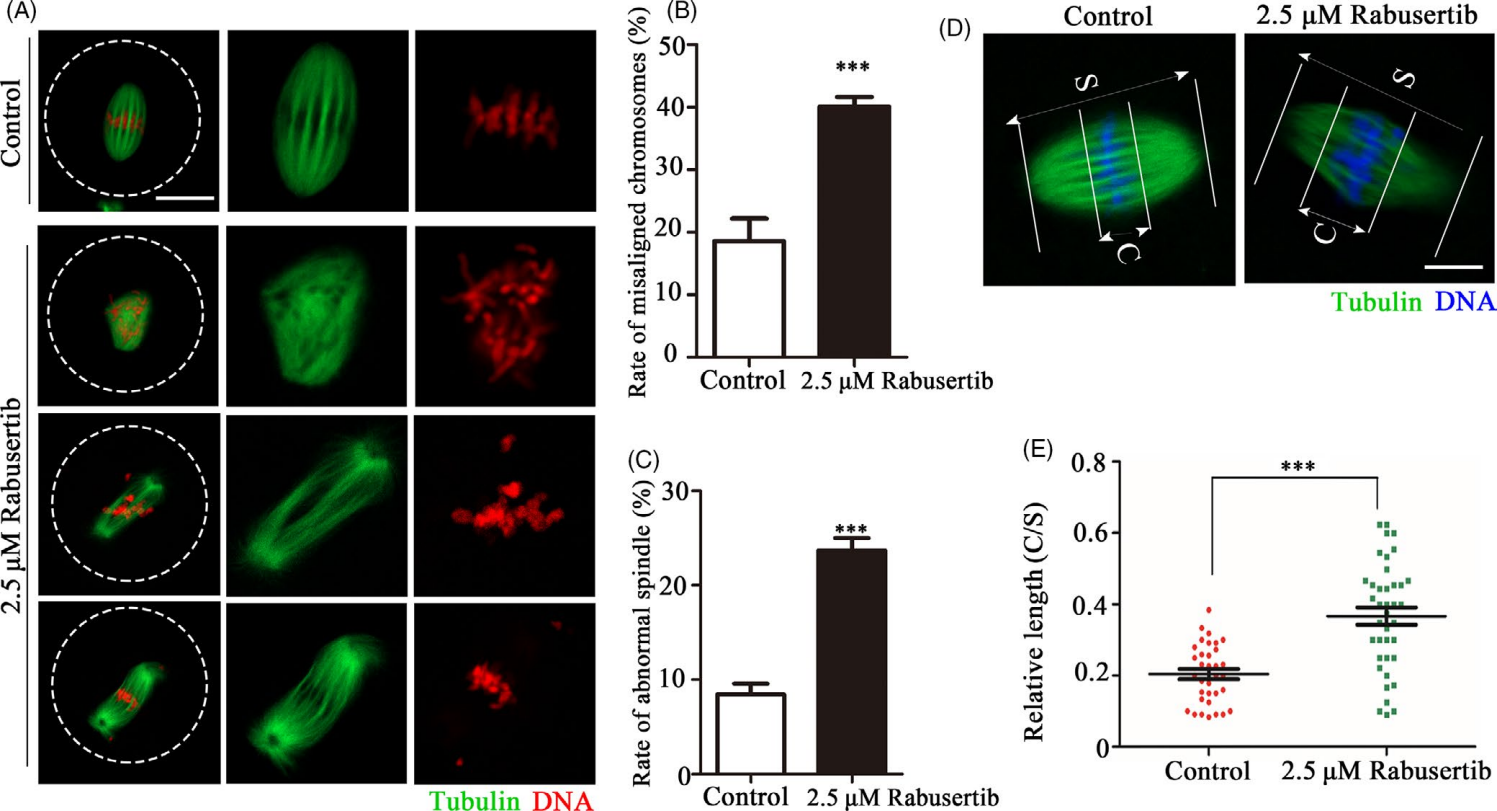
Inhibiting checkpoint kinase 1 (CHK1) affects spindle morphology and chromosome alignment at the first cleavage of early mouse embryos. A, Spindle morphology and chromosome alignment were disturbed after inhibiting CHK1. The control and CHK1‐inhibited embryos at metaphase were stained with the anti‐α‐tubulin (green) and counterstained with Hoechst 33342 to visualize the chromosomes (red). Bar = 20 μm. B, The incidence of spindle defects in the treatment and control embryos. ***Significant difference (*P* < .001). C, The incidence of chromosome misalignment in the treatment and control embryos. ***Significant difference (*P* < .001). D, Measurements of spindle middle plate thickness. C indicates maximum span of the chromosomes. S indicates maximum spindle length. Bar = 10 μm. E, Scattergram shows the C:S ratios for the treatment and control embryos. ***Significant difference (*P* < .001)

We observed the spindle assembly and chromosome morphology during the first cleavage process to explain how CHK1 regulates embryonic development. The results showed that chromosome alignment and spindle organization were disturbed at the first cleavage of embryos in the treatment group compared with the control group, revealing lagging or scattered chromosomes and multipolar/unipolar spindles (Figure 2A). The spindle abnormality rate was significantly higher in the treatment group of embryos than in the control group of embryos (40.03 ± 2.8%, n = 56 vs 18.62 ± 6.34%, n = 68, *P* < .01; Figure 2B). Similarly, the incidence of chromosomal misalignment was higher in the treatment group of embryos than in the control groups of embryos (23.72 ± 2.28%, n = 60 vs 8.43 ± 1.96%, n = 52, *P* < .001; Figure 2C). We measured the width of the spindle plate to quantitatively evaluate the degree of chromosomal abnormality in the embryos. That is, the area occupied by the spindle‐shaped plate relative to spindle length (Figure 2D). The results showed that the width of the plate in the treatment group embryos was significantly larger than that of control group embryos (0.37 ± 0.15, n = 39 vs 0.20 ± 0.08, n = 34, *P* < .001; Figure 2E). Overall, these results indicated that inhibiting CHK1 caused severely abnormal spindle assembly and chromosomal misalignment.

### 3.3 Inhibiting CHK1 affects kinetochore‐microtubule attachment and localization of BubR1 and Aurora B in mouse embryos

**Figure 3:**
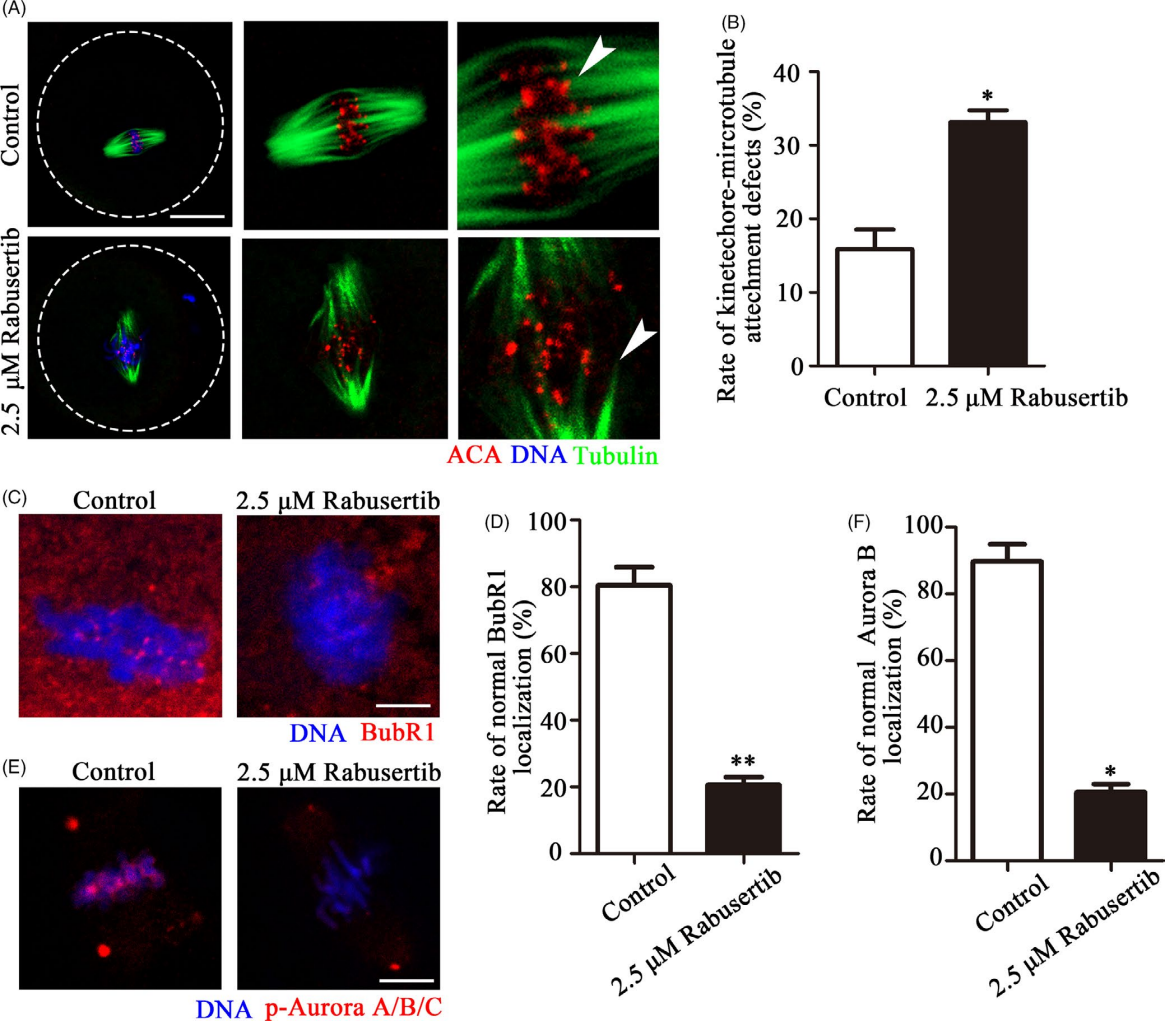
Inhibiting checkpoint kinase 1 (CHK1) affects kinetochore‐microtubule attachment and location of the spindle assembly checkpoint (SAC) proteins. A, Unattached microtubules were found in the treatment group embryos after the cold treatment. Control and CHK1 inhibited embryos were stained with an anti‐α‐tubulin antibody to visualize the spindle (green) and with ACA to visualize the kinetochores (red). Embryos were counterstained with DAPI to visualize the chromosomes (blue). Bar = 20 μm. B, The incidence of kinetochore‐microtubule attachment defects in the control and CHK1 inhibited embryos. *Significant difference (*P* < .05). C, Control and CHK1‐inhibited embryos were stained with a BubR1 antibody to recognize BubR1 (red). Embryos were counterstained with DAPI to visualize chromosomes (blue). Bar = 5 μm. D, Localization of BubR1 in the treatment and control embryos. **Significant difference (*P* < .01). E, Control and CHK1‐inhibited embryos were stained with DAPI to visualize the chromosomes and with anti‐p‐Aurora A/B/C to recognize Aurora B (red). Bar = 10 μm. F, Localization of Aurora B in the treatment and control embryos. *Significant difference (*P* < .05)

Due to the chromosomal misalignment observed after inhibiting CHK1 activity, we explored whether this phenomenon was related to kinetochore‐microtubule attachment, as this determines activity of the SAC. We evaluated the stability of the kinetochore‐microtubule attachment by cold treatment to disaggregate the unstable microtubules that were not attached to kinetochores. As shown in Figure 3A, the kinetochores were clearly connected to the microtubules in the control embryos, while some of the kinetochores were unable to match the corresponding microtubules in the treatment group embryos. The ratio of cells with unattached kinetochores was significantly higher in the treatment group than that in the control group (33.15 ± 3.21%, n = 48 vs 15.84 ± 5.39%, n = 35, *P* < .05; Figure 3B). To further confirm that CHK1 is involved in regulation of the SAC to accelerate the cell cycle process, we verified the relationship between CHK1, the SAC protein BubR1 and the checkpoint‐related protein Aurora B. The location of BubR1 was lost on metaphase kinetochores in the treatment group compared with the control group (Figure 3C). About 80% of the embryos showed lost BubR1 signals at the kinetochores in the treatment group (Figure 3D). A similar finding was observed for the p‐Aurora B signal (20.56 ± 2.42%, n = 28 vs 89.68 ± 5.20%, n = 24, *P* < .05; Figure 3E,F). These results suggested that CHK1 participated in control of the SAC by affecting recruitment of BubR1 and Aurora B, which further caused kinetochore‐microtubule attachment defects during the first embryonic cleavage.

### 3.4 Inhibiting CHK1 induces DNA damage and oxidative stress during early mouse embryonic development

**Figure 4:**
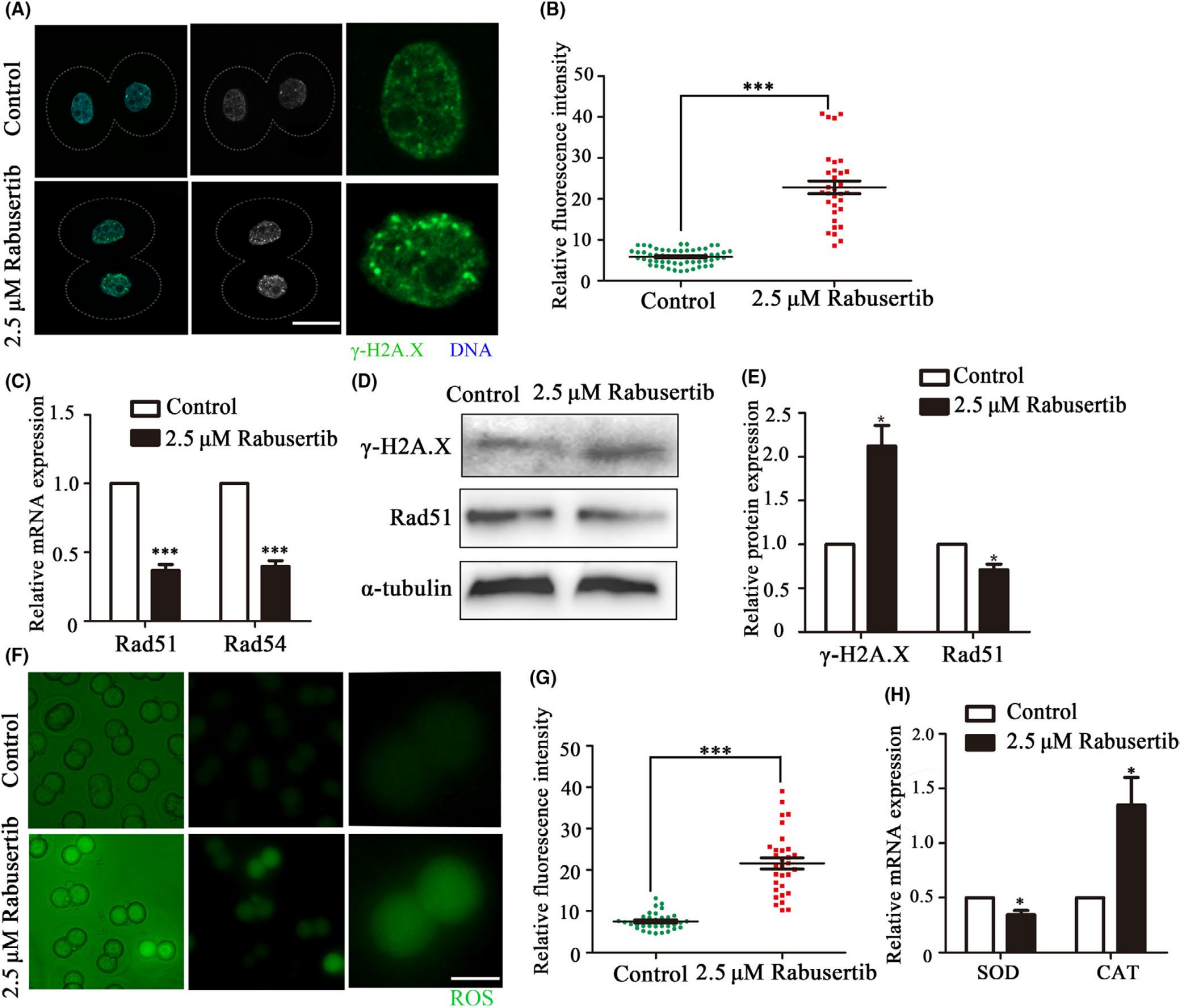
Inhibiting checkpoint kinase 1 (CHK1) affects DNA damage and oxidative stress during early mouse embryonic development. A, The control and CHK1 inhibited embryos at the 2‐cell stage were stained with anti‐γ‐H2A.X (green). Bar = 20 μm. B, γ‐H2A.X fluorescence intensity in the treatment and control group embryos. ***Significant difference (*P* < .001). C, The expression of DNA damage response (DDR)‐related genes in the treatment and control groups. ***Significant difference (*P* < .001). D, The protein expression levels of Rad51 and γ‐H2A.X in embryos in the control and treatment groups were determined by immunoblotting. E, Band intensity analysis of Rad51 and γ‐H2A.X in the two groups. *Significant difference (*P* < .05). F, The control and CHK1‐inhibited embryos were stained for reactive oxygen species (ROS) (green) at the 2‐cell stage. Bar = 50 μm. G, The fluorescence intensity of ROS in the treatment and control embryos. ***Significant difference (*P* < .001). H, The expression of ROS‐related genes in the treatment and control groups. *Significant difference (*P* < .05)

To explore the causes for the morula/blastocyst defects after inhibiting CHK1, we used γ‐H2A.X as a marker protein to detect the effects of CHK1 on interphase DNA damage. The immunofluorescence staining results showed that the γ‐H2A.X protein was highly enriched in chromatin in the treatment group embryos compared with the control embryos (Figure 4A). γ‐H2A.X fluorescence intensity in the treatment group embryos was higher than that of the control group embryos (22.84 ± 8.85, n = 59 vs 5.87 ± 1.82, n = 32, *P* < .0001; Figure 4B). Moreover, DNA damage response‐related gene expression was detected by RT‐PCR. The expression levels of Rad51 and Rad54 decreased significantly in the treatment group compared with the control group (Rad51, 1.00 vs 0.37 ± 0.04, *P* < .001; Rad54, 1.00 vs 0.40 ± 0.04, *P* < .001, Figure 4C). We also examined γ‐H2A.X and Rad51 protein expression, and the results showed that γ‐H2A.X expression increased, while Rad51 expression decreased in the CHK1 inhibition group (Figure 4D). The band intensity analysis also confirmed this finding (γ‐H2A.X: 1 vs 2.12 ± 0.24, *P* < .05; Rad51: 1 vs 0.71 ± 0.06, *P* < .05; Figure 4E).

As DNA damage can cause oxidative stress, we explored whether disrupting CHK1 activity affected the ROS level in early mouse embryos. The results indicated that ROS levels increased in early embryos after inhibiting CHK1 activity (Figure 4F). The fluorescence intensity of ROS in the embryos of the treatment group was significantly higher than that of embryos in the control group (21.62 ± 7.62, n = 31 vs 7.56 ± 2.12, n = 32, *P* < .0001; Figure 4G). In addition, we analysed the expression of genes related to oxidative stress by RT‐PCR. Catalase (CAT) expression levels in the treatment group (1.00 vs 2.699 ± 0.228, *P* < .05) and superoxide dismutase (SOD) (1.00 vs 0.693 ± 0.172, *P* < .01) (Figure 4H) were significantly disrupted compared with those in the control group. These results suggested that inhibiting CHK1 induced DNA damage and oxidative stress in early mouse embryos.

### 3.5 Inhibiting CHK1 induces early apoptosis in mouse embryos

**Figure 5:**
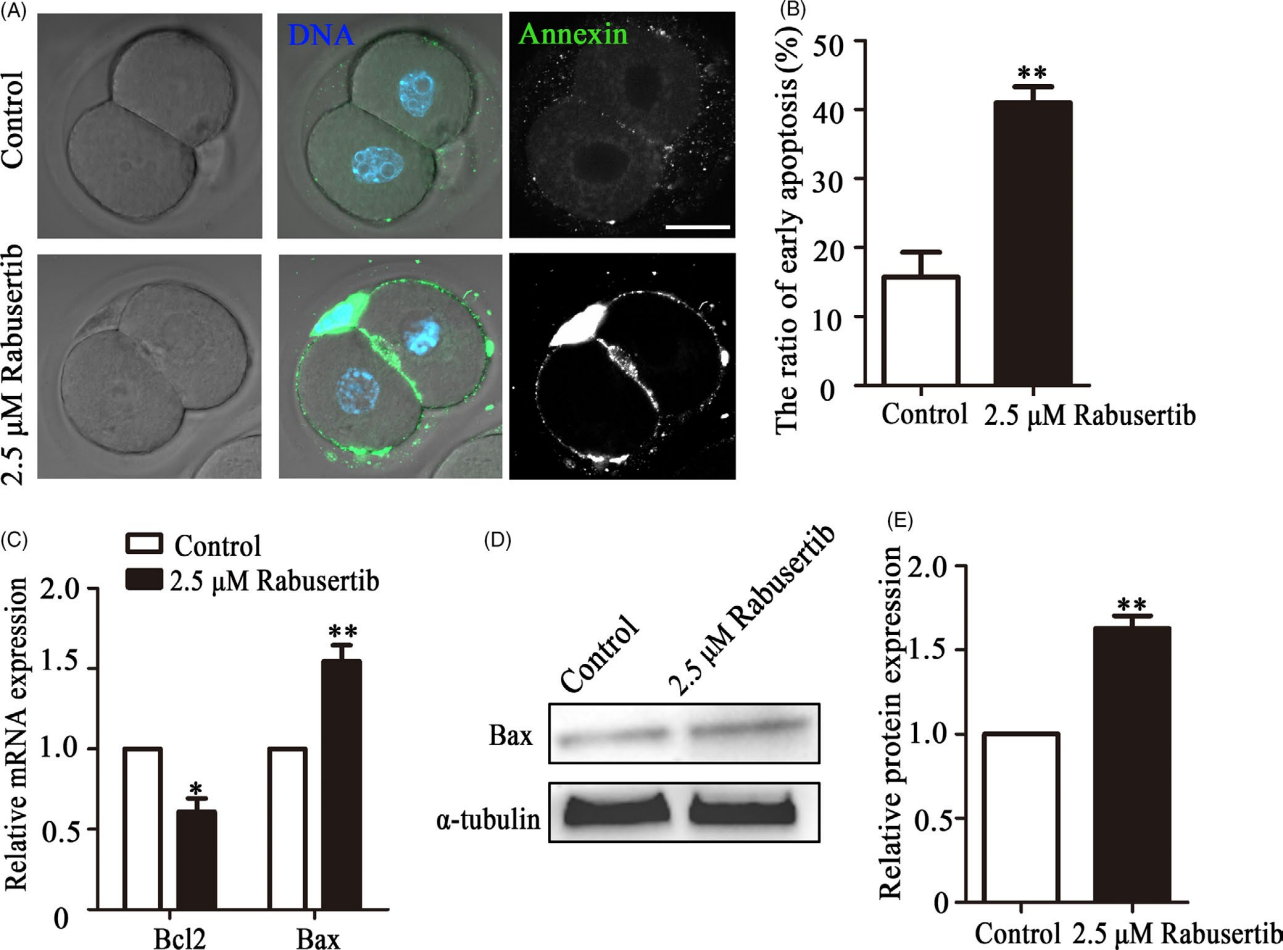
Inhibiting checkpoint kinase 1 (CHK1) induces apoptosis during early embryonic development in mice. A, The control and CHK1‐inhibited embryos at the 2‐cell stage were stained with Annexin‐V (green). Bar = 30 μm. B, The percentages of cells in early apoptosis in the mouse embryo treatment and control groups. **Significant difference (*P* < .01). C, The expression of apoptosis‐related genes in the treatment and control groups. **Significant difference (*P* < .01). *Significant difference (*P* < .05). D, Bax expression in embryos from the control and treatment groups was determined by immunoblotting. E, Band intensity analysis of Bax in the two groups. **Significant difference (*P* < .01)

We next performed Annexin‐V staining of embryos to check for early apoptosis. Our results revealed Annexin‐V‐positive signals in the treatment embryos, indicating early apoptosis (Figure 5A). The percentage of apoptosis‐positive embryos was significantly higher in the treatment group than that in the control group. (40.98 ± 2.35, n = 46, vs 15.73 ± 3.59, n = 48, *P* < .001, Figure 5B). Furthermore, an examination of the expression of apoptotic genes showed that relative Bax mRNA expression was upregulated (1.55 ± 0.10 vs 1.00, n = 90, *P* < .01), whereas Bcl2 expression was downregulated (0.61 ± 0.08 vs 1.00, n = 90, *P* < .05) in the treatment group (Figure 5C). We also assessed Bax protein expression to further confirm the effects of the CHK1 on apoptosis, and the results showed that Bax expression increased in the CHK1 inhibited group (Figure 5D). A band intensity analysis confirmed this finding (1.63 ± 0.08 vs 1.00, *P* < .01, Figure 5E). These data indicated that loss of CHK1 activity induced early apoptosis in early embryos.

### 3.6 Inhibiting CHK1 induces autophagy in early mouse embryos

**Figure 6:**
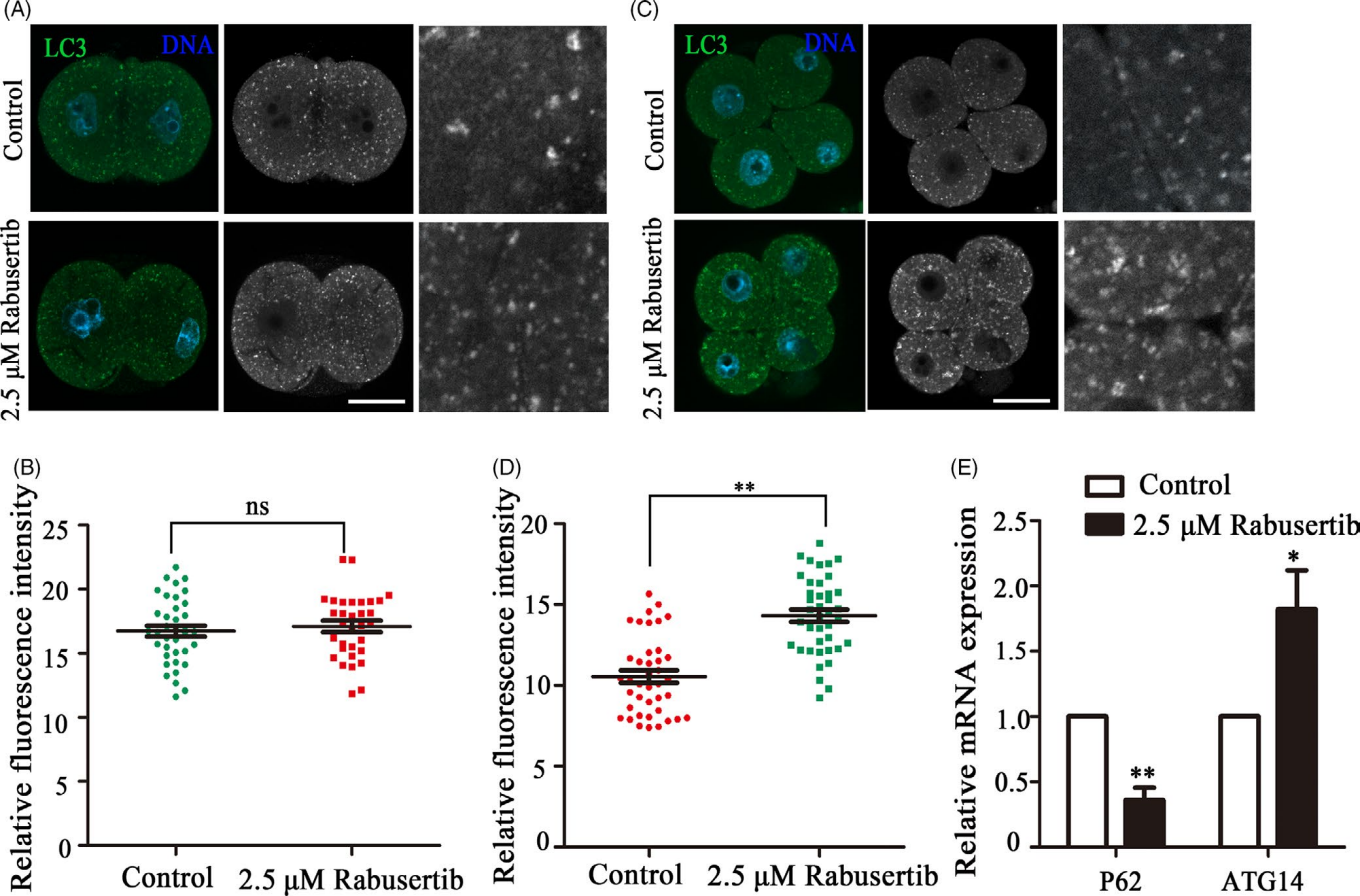
Inhibiting checkpoint kinase 1 (CHK1) induces autophagy in mice during early embryonic development. A, The control and CHK1‐inhibited embryos were immunolabeled with anti‐LC3 antibody (green) at the 2‐cell stage. Hoechst 33342 was used to label DNA (blue). Bar = 30 μm. B, LC3 fluorescence intensity of the treatment and the control embryos at the 2‐cell stage. C, Control and CHK1‐inhibited embryos were immunolabeled at the 4‐cell stage with anti‐LC3 antibody (green), and Hoechst 33342 was used to label DNA (blue). Bar = 30 μm. D, LC3 fluorescence intensity of the treatment and control embryos at the 4‐cell stage. **Significant difference (*P* < .01). E, The expression of autophagy‐related genes in the treatment and control groups. **Significant difference (*P* < .01). *Significant difference (*P* < .05)

We also collected mouse embryos at the 2‐cell and 4‐cell stages to examine autophagy status by LC3 staining. No significant change in LC3 immunofluorescence intensity was detected in 2‐cell embryos between the control and treatment groups (16.74 ± 2.59, n = 37 vs 17.50 ± 2.10, n = 32, Figure 6A,B). However, more autophagic vesicles than normal 4‐cell embryos were observed in the CHK1‐inhibited 4‐cell embryos (Figure 6C). Relative LC3 fluorescence intensity was higher in the treated embryos than that in the control embryos (10.54 ± 0.38, n = 40 vs 14.31 ± 0.38, n = 40, *P* < .01; Figure 6D). In addition, the expression levels of the autophagy‐related genes ATG14 (1.00 vs 1.82 ± 0.66, *P* < .05) and P62 (1.00 vs 0.36 ± 0.16, *P* < .05; Figure 6E) were significantly different in the treatment group from those in the control group. These results indicated that inhibiting CHK1 activity caused autophagy in early mouse embryos.

## 4 DISCUSSION

**Figure 7:**
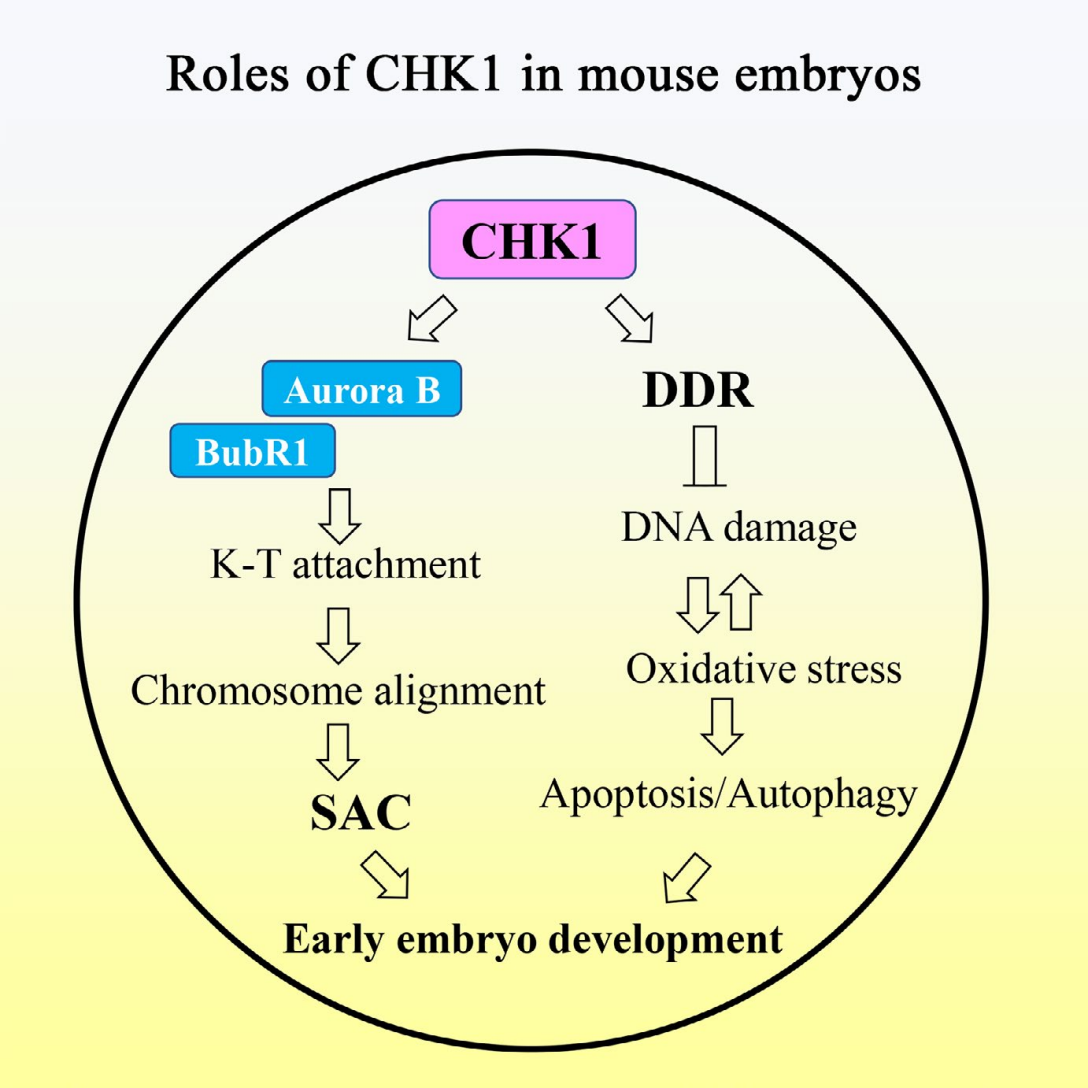
Diagram of the roles of checkpoint kinase 1 (CHK1) during mouse early embryonic development. CHK1 play roles in both DNA repair and the spindle assembly checkpoint (SAC) during early cleavage of mouse embryos

We used mice as a model to study the functions of CHK1 during embryonic cleavage. Our results indicated that CHK1 participated in control of the SAC and DNA damage repair in mouse embryos through its effects on BubR1/Aurora B recruitment for kinetochore‐microtubule attachment and control of intracellular oxidative stress, apoptosis and autophagy (Figure 7).

CHK1 is a central mediator of the DDR at the S and G2/M cell cycle checkpoints and plays a crucial role preserving genomic integrity.^16^ However, the regulation of CHK1 during early embryonic cleavage remains unclear. Our results showed that loss of CHK1 activity accelerated the cell cycle during the first cleavage of mouse embryos. However, interestingly it delayed/disturbed cleavage after prolonged embryonic culture, indicating that CHK1 was involved in the regulation of mouse early embryonic cleavage. The precocious cleavage to a 2‐cell embryo was similar to depletion of the SAC protein phenotypes (such as Bub3, BubR1 and Mad2) in early mouse embryos.^12^ Previous studies have shown that activating DNA damage checkpoints promotes function of the SAC and that CHK1 participates in regulating SAC abnormal mitotic processes.^21^ Therefore, we speculated that CHK1 might be involved in regulating the SAC during the first cleavage of mouse embryonic development, and loss of CHK1 activity caused aberrant misaligned chromosomes, which was similar with studies in mouse and pig oocytes.^25^, ^26^ The spindle assembly checkpoint monitors microtubule attachment and tension to ensure the fidelity of chromosome segregation during mitosis.^27^ Our results showed that inhibiting CHK1‐induced defects of kinetochore‐microtubule attachment, which was consistent with a previous study reporting that CHK1 and Mps1 jointly regulate the correction of merotelic kinetochore attachments.^24^

Next, we examined localization of BubR1 and Aurora B to explore how CHK1 regulates kinetochore‐microtubule attachment and SAC activity, as CHK1 is reported to be essential for recruiting BubR1 to the kinetochores,^28^ while CHK1 associates with Aurora B for post‐mitotic genome surveillance of cytokinetic abscission.^29^ Our results showed that BubR1 and Aurora B failed to locate to the kinetochores without CHK1 activity, which was consistent with previous studies. Taken together, these data showed that CHK1 recruited BubR1/Aurora B for kinetochore‐microtubule attachment and chromosome alignment during the first cleavage of mouse embryonic development, which might be the cause for the accelerated cell cycle during this process.

Cells have a highly organized and coordinated mechanism to ameliorate genotoxic stress called the DDR.^30^ ATR is the apical kinase that responds to DNA damage.^31^ CHK1 kinase is the downstream effector of ATR that delays the cell cycle and stabilizes the replication fork by controlling replication origin firing, creating a time window to correct DNA damage and ensuring that cells cannot enter mitosis when replication is incomplete.^32^, ^33^ Some reports indicate that CHK1 kinase is an important DNA damage checkpoint component and that its loss will cause cell cycle arrest.^34^, ^35^ We examined whether CHK1 monitors DNA damage in the mouse embryo model, and the results showed that inhibiting CHK1 led to an increase in γ‐H2A.X fluorescence intensity and a decrease of DDR‐related gene Rad51 and Rad54 expression. γ‐H2A.X is an important readout for initializing the DNA damage checkpoint and successful sensing of DNA damage,^36^ and Rad51 and Rad54 are involved in DNA double‐strand break repair and recombination,^37^ indicating that CHK1 is essential for DNA damage repair in early embryos. While DNA damage increases the level of ROS and induces oxidative stress,^38^, ^39^ our results showed that inhibiting CHK1 also increased ROS production, confirming DNA damage. In addition, DNA damage and oxidative stress usually activate apoptosis and autophagy to clear the damaged cells. It has been reported that DNA damage leads to autophagy through ATR/CHK1/RhoB‐mediated lysosomal recruitment of the TSC complex and subsequent inhibition of mTORC1.^40^ CHK1 is activated by caspase‐mediated cleavage during apoptosis and might be implicated in enhancing apoptotic reactions.^41^ Our results also showed that inhibiting CHK1 enhanced the apoptotic signal and increased the number of intracellular autophagic vesicles in early mouse embryos. In addition, Bax protein expression increased, while expression of the autophagy‐related genes ATG14 and P62 was altered, indicating the occurrence of apoptosis and autophagy. The pro‐apoptotic BCL‐2 proteins commit cells to apoptosis. Bax is a key component of cellular‐induced apoptosis through mitochondrial stress,^42^ indicating that inhibiting CHK1 activity lead to apoptosis by downregulating Bcl‐2 and upregulating Bax. Next, we tested the autophagy‐related genes P62 and ATG14. ATG14 and P62 participate in important pathways as key autophagy proteins.^43^ Similar to the increasing trend of apoptosis, inhibiting CHK1 activity affected expression of the ATG14 and P62 genes and induced autophagy. These results indicated that CHK1 was involved in DNA damage repair, which protected against oxidative stress and early apoptosis, and might be the cause for the subsequent cleavage defects after the 2‐cell stage in early mouse embryos.

In summary, our results indicated that CHK1 was critical for mouse early embryonic development through its double roles as a regulator of DNA damage checkpoints and control of the SAC.

## CONFLICTS OF INTEREST

No conflicts of interest are declared.

## AUTHOR CONTRIBUTION

JQJ and SCS conceptualized and designed the study. JQJ acquired the data. JQJ and SCS analysed and interpretation of the data. XHL, MHP, YX, YX and MHS contributed to materials and reagents. JQJ drafted the article. All authors have approved the final version of the submitted manuscript.

## Data Availability

All data are in the manuscript.
